# Supplementary material for: A fault-intrusion-tolerant system and deadline-aware algorithm for scheduling scientific workflow in the cloud
Source: PeerJ Comput Sci. 2021 Nov 2;7:e747. doi: 10.7717/peerj-cs.747 (PMC8576554; doi:10.7717/peerj-cs.747)
Supplement: Supplemental Information 6 [file peerj-cs-07-747-s006.zip › Myproject1.1/docs/org/cloudbus/cloudsim/power/class-use/PowerDatacenter.html]

Uses of Class org.cloudbus.cloudsim.power.PowerDatacenter


JavaScript is disabled on your browser.


- Overview
- Package
- Class
- Use
- Tree
- Deprecated
- Index
- Help

- Prev
- Next

- Frames
- No Frames

- All Classes

## Uses of Class org.cloudbus.cloudsim.power.PowerDatacenter

- Packages that use PowerDatacenter

  | Package | Description |
  | --- | --- |
  |  |  |
  | --- | --- |
  | org.cloudbus.cloudsim.examples.power |  |
  | org.cloudbus.cloudsim.power |  |
- - ### Uses of PowerDatacenter in org.cloudbus.cloudsim.examples.power

    Methods in org.cloudbus.cloudsim.examples.power with parameters of type PowerDatacenter

    | Modifier and Type | Method and Description |
    | --- | --- |
    |  |  |
    | --- | --- |
    | `static void` | Helper.`printResults(PowerDatacenter datacenter, java.util.List<Vm> vms, double lastClock, java.lang.String experimentName, boolean outputInCsv, java.lang.String outputFolder)` Prints the results. |
  - ### Uses of PowerDatacenter in org.cloudbus.cloudsim.power

    Subclasses of PowerDatacenter in org.cloudbus.cloudsim.power

    | Modifier and Type | Class and Description |
    | --- | --- |
    |  |  |
    | --- | --- |
    | `class` | `PowerDatacenterNonPowerAware` PowerDatacenterNonPowerAware is a class that represents a non-power aware data center in the context of power-aware simulations. |

- Overview
- Package
- Class
- Use
- Tree
- Deprecated
- Index
- Help

- Prev
- Next

- Frames
- No Frames

- All Classes
